# Effect of different phosphate sources on uranium biomineralization by the *Microbacterium* sp. Be9 strain: A multidisciplinary approach study

**DOI:** 10.3389/fmicb.2022.1092184

**Published:** 2023-01-09

**Authors:** Pablo Martínez-Rodríguez, Iván Sánchez-Castro, Jesús J. Ojeda, María M. Abad, Michael Descostes, Mohamed Larbi Merroun

**Affiliations:** ^1^Department of Microbiology, University of Granada, Granada, Spain; ^2^Department of Chemical Engineering, Faculty of Science and Engineering, Swansea University, Swansea, United Kingdom; ^3^Centro de Instrumentación Científica (CIC), University of Granada, Granada, Spain; ^4^Environmental R&D Department, ORANO Mining, Chatillon, France; ^5^Centre de Géosciences, MINES Paris, PSL University, Fontainebleau, France

**Keywords:** *Microbacterium*, uranium, phosphate source, bioprecipitation, solubilization, PSB

## Abstract

**Introduction:**

Industrial activities related with the uranium industry are known to generate hazardous waste which must be managed adequately. Amongst the remediation activities available, eco-friendly strategies based on microbial activity have been investigated in depth in the last decades and biomineralization-based methods, mediated by microbial enzymes (e.g., phosphatase), have been proposed as a promising approach. However, the presence of different forms of phosphates in these environments plays a complicated role which must be thoroughly unraveled to optimize results when applying this remediation process.

**Methods:**

In this study, we have looked at the effect of different phosphate sources on the uranium (U) biomineralization process mediated by *Microbacterium* sp. Be9, a bacterial strain previously isolated from U mill tailings. We applied a multidisciplinary approach (cell surface characterization, phosphatase activity, inorganic phosphate release, cell viability, microscopy, etc.).

**Results and Discussion:**

It was clear that the U removal ability and related U interaction mechanisms by the strain depend on the type of phosphate substrate. In the absence of exogenous phosphate substrate, the cells interact with U through U phosphate biomineralization with a 98% removal of U within the first 48 h. However, the U solubilization process was the main U interaction mechanism of the cells in the presence of inorganic phosphate, demonstrating the phosphate solubilizing potential of the strain. These findings show the biotechnological use of this strain in the bioremediation of U as a function of phosphate substrate: U biomineralization (in a phosphate free system) and indirectly through the solubilization of orthophosphate from phosphate (P) containing waste products needed for U precipitation.

## 1. Introduction

Uranium is a naturally occurring element that is ubiquitous in the Earth’s crust (e.g., soil and water). However, anthropogenic activities related to uranium mining, the nuclear energy industry, or weapon manufacturing may involve the redistribution of this element in the environment, sometimes resulting in locally high U concentrations (Kolhe et al., 2018). Due to U radiological and chemical toxicity, remediation strategies are highly recommended to prevent the potentially harmful effects for the environment and health.

Several remediation approaches have been proposed to remove inorganic contaminants from polluted sites leading to a decrease in their associated risks (Gavrilescu et al., 2009). Traditional strategies based on physico-chemical methods, such as precipitation, evaporation, or extraction amongst others, have been used to treat heavy-metal contaminated sites (Selvakumar et al., 2018). However, these approaches are known to be costly at environmental level in comparison with biologically based remediation. Both types of techniques are often additionally used for more efficient and economical rehabilitation of contaminated areas (Khalid et al., 2017). In recent years, a wide range of microorganisms isolated from contaminated sites (such as mining areas) have been shown to possess the ability to immobilize U (Banala et al., 2021). The main strategies which have been adopted by microorganisms to avoid U cytotoxic effects are based on different mechanisms such as U biosorption at the cell surface (Merroun et al., 2006), U biomineralization (Macaskie et al., 2000; Beazley et al., 2007; Lopez-Fernandez et al., 2021), U intracellular accumulation (Merroun et al., 2003; Gerber et al., 2016) and U biotransformation, either by reduction (Lovley et al., 1991; Williams et al., 2013) or by oxidation (DiSpirito and Tuovinen, 1982). In particular, one of the most promising approaches consists of U-biomineralization under aerobic conditions (Beazley et al., 2011; Tu et al., 2019; Sánchez-Castro et al., 2020).

The uranium biomineralization process probably occurs in the presence of ligands such as carbonates, phosphates and hydroxides, which act as nucleation sites generated by microbial activity (Lloyd and Macaskie, 2000; Wufuer et al., 2017). Amongst the different mineralization processes, U-phosphate precipitation has been well documented in recent years by numerous studies (Zhang et al., 2020). They are generally bi-phasic processes based on passive U binding at the cell surface through phosphate and/or carboxyl groups and a metabolism-dependent release of orthophosphates from organic phosphate substrates [e.g., glycerol-2-phosphates (G2P), glycerol-3-phosphates (G3P)] mediated by phosphatase activity (Lopez-Fernandez et al., 2021). The resulting orthophosphates interact with uranyl ion (UO_2_^2+^), the most common soluble form of environmental U, making it immobile and consequently less toxic. Specifically, meta-autunite-like U phosphate forms have been widely studied due to their long-term stability under different physico-chemical conditions (Beazley et al., 2009). These U-phosphate compounds are attractive for bioremediation purposes rather than the uraninite forms produced during bioreduction processes which are known to be less stable and easily oxidized (Wang et al., 2013). Previous studies have reported the formation of U phosphates in presence of determined microbial strains and organic phosphate sources such as G3P (Beazley et al., 2011) or G2P (Macaskie et al., 2000; Newsome et al., 2015; Sánchez-Castro et al., 2020), as well as inorganic phosphates (Pi). The microbial-mediated formation of U phosphates is known to be affected by different physico-chemical factors such as pH (Zheng et al., 2018), redox conditions (Salome et al., 2013), co-existing cations/anions (Wei et al., 2019) and humic substances (Boiteau et al., 2018), which may decrease the bioprecipitation efficiency. However, to the best of our knowledge, no studies on the impact of different forms of phosphates (organic/inorganic) on U biomineralization have been conducted so far. The presence of different phosphate forms should be considered, especially when phosphate solubilizing bacteria (PSB) are used as bioremediation agents or are present in the medium. PSB are known to play a major role in enhancing phosphate-induced immobilization of heavy metals such as U for bioremediation purposes (Sowmya et al., 2014). However, eutrophication issues should be contemplated since there is a limit on the amount of phosphate which can be added to the environment (Park et al., 2011). Therefore, the use of PSB (e.g., the species of the genus *Microbacterium*) in the bioremediation of heavy metals could contribute to the circular economy by using cheap and readily available material which is rich in insoluble phosphates.

This study addresses the impact of phosphate forms (organic/inorganic) in U biomineralization by *Microbacterium* sp. Be9, a bacterial strain isolated from U mill tailings. In previous studies, this bacterium showed high tolerance to different heavy metals (Sánchez-Castro et al., 2017). Moreover, members of the *Microbacterium* genus have been described for their potential role in uranium-contaminated water remediation. They have been shown to accumulate uranium both at cell surface level and also extracellularly as uranium phosphates at pH 4.5 (Nedelkova et al., 2007) displaying multiple detoxification mechanisms involving phosphates (Theodorakopoulos et al., 2015). According to the latter works, *Microbacterium* sp. strain Be9 would display similar U interaction mechanisms mediated by different enzymatic activities upon phosphate source (phosphatase in presence of organic phosphates and polyphosphatase in a free phosphate system) leading to the biomineralization of U as U phosphate mineral phases. Thus, Be9 could precipitate U under the presence of organic phosphates. Bacterial strains with phosphatase activity are capable of releasing orthophosphates from organophosphate sources in order to avoid the stress caused by U. In presence of inorganic phosphates, most of the U should remain in the insoluble form. In our study, systems with three different sources of phosphate were assayed (no-phosphate, organic and inorganic phosphates) to investigate the mechanisms displayed by the Be9 strain under U presence. We have used a multidisciplinary approach combining colorimetric methods for measuring residual uranium and inorganic-phosphate release, as well as flow cytometry to assess bacterial activity and viability. A characterization of Be9 membrane surface was determined by potentiometric titrations and X-ray photoelectron spectroscopy (XPS) analysis. Moreover, we also used microscopic and spectroscopic techniques [Scanning Transmission Electron Microscopy-High Angle Annular Dark-Field (STEM-HAADF) and X-ray Diffraction (XRD)] to localize at cellular level the U precipitates obtained and to analyze their composition.

## 2. Materials and methods

### 2.1. Bacterial culture

*Microbacterium* sp. strain Be9 was isolated from mill-tailing repository sites, located near Bessines-sur-Gartempe (Limousin, France; Sánchez-Castro et al., 2017). The cultures were maintained and grown in a solid/liquid Lysogeny broth (LB) medium (tryptone 10 g/l, yeast extract 5 g/l and NaCl 10 g/l, pH 7.0 ± 0.2) at 28°C with shaking at 160 rpm. For long-term storage, the cultures were stored at −80°C in 50% glycerol.

### 2.2. Potentiometric titrations

Potentiometric titrations were carried out to determine the chemical properties of *Microbacterium* sp. Be9 cell surface. An amount of live bacteria equivalent to 0.14–0.19 g of dry biomass (washed four times with NaClO_4_) was suspended in a vessel with 20 ml of 0.1 M NaClO_4_. The suspension was titrated with 0.1 M HCl to pH 3.5 followed by 0.1 M NaOH to pH 10.0. To test the reversibility of the protonation–deprotonation behavior, the suspension was back-titrated with 0.1 M HCl from pH 10.0 to 3.5. HCl and NaOH solutions were previously calibrated against primary standards. All the titrations were performed using a Metrohm^®^ *Titrando 906* automatic titrator (Metrohm, United Kingdom) at 25°C. The temperature was kept constant and continuously monitored during the titration. To calculate the acidity constant (pKa) values and the corresponding total concentration of the binding sites for Be9 cells, data from three replicates of each titration curve were fitted using the program Protofit 2.1 rev1 (Turner and Fein, 2006) using a Non-Electrostatic Model (NEM).

### 2.3. X-ray photoelectron spectroscopy

To characterize the elemental composition of the near-cell surface of Be9 strain (2–5 nm penetration depth), the X-ray photoelectron spectroscopy technique was used. Be9 pellets were freeze-dried and the obtained powder was mounted on standard studs using double-sided adhesive tape. XPS measurements were made on a Kratos Supra photoelectron spectrometer at 10 kV and 20 mA using a monochromatic Al Kα X-ray source (1486.6 eV). The take-off angle was fixed at 90°. For each sample the data were collected from three randomly selected locations, and the area corresponding to each acquisition was 400 μm in diameter. Each analysis consisted of a wide survey scan (pass energy 160 eV, 1.0 eV step size) and high-resolution scan (pass energy 20 eV, 0.1 eV step size) for component speciation. The binding energies of the peaks were determined using the C_1s_ peak at 284.5 eV. To fit the XPS spectra peaks CasaXPS software (version 2.3.22PR1.0)^1^ was used (Fairley, 2020).

### 2.4. Uranium speciation modelling

Uranium speciation in different assayed media (organic and inorganic phosphate sources) was determined by Visual MINTEQ version 3.1^2^ and PhreeqC software (calculated using PRODATA thermodynamic database). The initial conditions of temperature, pH and U concentration were incorporated into the model as 28°C, 6.6 and 100 μM, respectively.

### 2.5. Phosphate impact on U biomineralization: Experimental design

The *Microbacterium* sp. strain Be9 was grown in liquid LB medium in continuous shaking (160 rpm) at 28°C for 24 h. The cells were harvested by centrifugation for 5 min at 10,000 × *g* and washed twice with 0.9% NaCl solution to remove the interfering elements of the growth medium. In all cases, the acid-washed glass Erlenmeyer flasks were filled with the corresponding incubation solution. In treatments including the Be9 cells, an initial optical density (O.D.) of 0.7 (at 600 nm) was used. All the flasks were incubated under controlled temperature (28°C) with shaking (160 rpm) for 48 h. Uranium-free, no-bacteria and heat-killed-bacteria (incubated at 80°C for 1 h) flasks were considered as control treatments.

Potential U-bioprecipitation in presence of Be9 cells and organic and inorganic phosphate sources were investigated with the following conditions: (i) exogenous phosphate-free system (MOPS buffer + Be9 + U), labeled as MC1, (ii) organic phosphate (G2P) supplemented system (MOPS buffer + Be9 + G2P + U), labeled as MC2, and (iii) inorganic phosphate supplemented system (low phosphate medium, LPM + Be9 + U), labeled as MC3. Uranium was added as uranyl nitrate UO_2_ (NO_3_)_2_·6H_2_O from a 0.1 M stock solution to a final concentration of 100 μM, with the exception of the MC3 condition. In this case, the U concentration was increased to 0.5 and 1 mM due to the estimated presumption of abiotic U precipitation. To investigate the effect of the organic-phosphate source, flasks were filled with 20 mM 3-(N-Morpholino)propanesulfonic acid (MOPS; Sigma Aldrich) buffered at pH 6.5, and supplemented with 5 mM G2P (Sigma Aldrich). Control flasks without G2P were also tested. In the case of inorganic-phosphate sources, the culture medium LPM (Supplementary Table 1) at pH 7.5 was prepared as described in Lopez-Fernandez et al. (2018). In this condition, control flasks including reduced phosphate concentration (0.2 mg/l peptone) were also considered. Once the incubations were completed, aliquots from all the flasks were centrifuged at 10,000 × *g* for 10 min at 4°C, and the supernatants and solid pellets were analyzed separately. All the treatments were conducted in triplicate and the subsequent analyses used all the replicate data from each respective treatment for statistics.

### 2.6. Uranium removal quantification

Uranium concentrations in recovered supernatants were analyzed spectro-photometrically using the Arsenazo III method (Jauberty et al., 2013). The reagent was prepared by dissolving 70 mg of 2,7-bis (2-arsenophenylazo)-1,8-dihydroxynaphthalene-3,6-disulfonic acid (Arsenazo-III) into 1 l of 3 M HClO_4_. Then, 250 μl from each sub-sample were mixed with 1 ml of reagent. Absorbance was measured at 651 nm with Thermo Scientific^™^ GENESYS 10S UV–Vis spectrophotometer. Uranium removal in treatments was calculated as the difference between the initial and final U concentrations.

### 2.7. Inorganic phosphate quantification

After incubation, inorganic phosphate (Pi) concentration in supernatants was quantified by the ammonium-molybdate and ascorbic acid method (Murphy and Riley, 1962). This colorimetric procedure is based on the reaction of the orthophosphate ions with ammonium-molybdate in an acidic solution forming phosphomolybdic acid. After reduction with ascorbic acid, the resultant compound shows an intensely blue complex measurable at 850 nm after exactly 30 min.

### 2.8. Phosphatase activity

Phosphatase activity determination was carried out using Methylumbelliferyl (MUB)-linked phosphate as optimized and described (German et al., 2011). Be9 cells samples from different assays were dissolved in sodium perchlorate buffer (pH 5) and MUB standards (0.16, 0.625, 1.25, and 2.50 μM) in order to calculate the emission and quench coefficients using an automatic fluorometer NanoQuant Infinite 200 PRO (Tecan). Enzyme activity calculations were performed following German et al. (2011).

### 2.9. Bacterial viability and cell membrane potential

The cell viability and the cell membrane potential of *Microbacterium* sp. strain Be9 in the presence of U were determined through the flow cytometry technique. The cultures were prepared as stated above with an initial concentration of U of 100 μM. After 1, 24, and 48 h of incubation for viability, and 24 and 48 h for cell membrane potential, the cells were collected by centrifugation at 11,000 × *g* and 4°C for 10 min. The resultant pellet was washed three times and dissolved in phosphate buffered saline solution at pH 7. For the cell viability test, fluorescein diacetate and propidium iodide were added to each sample to a final concentration of 20 and 2 μl/ml, respectively. In the case of cell membrane potential, 3,3′-dihexyloxacarbocyanine iodide was used at a final concentration of 20 μl/ml. Finally, all the samples were analyzed by Forward Scatter using a FACSCanto II^™^ cytometer (Becton Dickinson, San Jose, CA, UnitedStates).

### 2.10. Microscopy analysis (STEM/HAADF)

Uranium-treated Be9 cells were harvested as described above, washed twice with 0.9% NaCl and sodium cacodylate buffer (pH 7.2), fixed with glutaraldehyde in a cacodylate buffer (4%) and stained with osmium tetraoxide (1%, for1 h) in the same buffer before being dehydrated through graded alcohol followed by propylene oxide treatment and finally embedded in epoxy resin. TEM specimen holders were cleaned by plasma prior to STEM analysis to minimize contamination. Ultrathin sections (0.1 μm) of the samples, obtained using an ultra-microtome, were loaded in a carbon coated copper grid. The samples obtained were observed by high-angle annular dark field scanning transmission electron microscope (HAADF-STEM), conducted using a FEI TITAN G2 80–300.

### 2.11. Statistical analysis

All the data were performed by GraphPad Prism version 8.0.0 for Windows (GraphPad Software, California, United States) and presented as averages and standard deviations for at least three replicates per experimental condition tested.

## 3. Results

### 3.1. Chemical characterization of the *Microbacterium* sp. Be9 cell surface

#### 3.1.1. Potentiometric titrations

**Figure 1:**
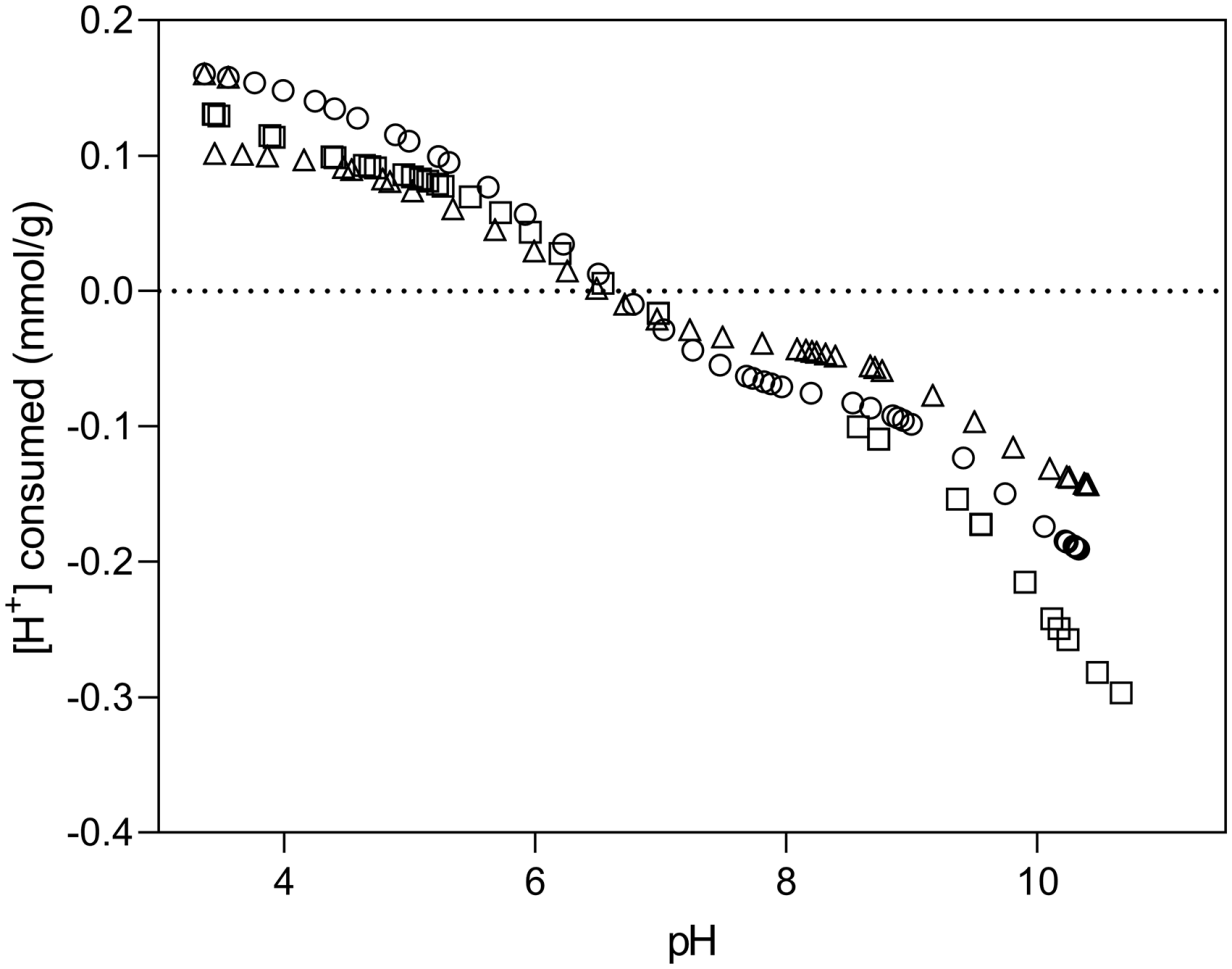
Potentiometric titration curves of Be9 strain in of 0.1 M NaClO_4_ at 25°C. The symbols refer to experimental replicates of each titration curve.

Potentiometric titration curves of *Microbacterium* sp. Be9 are presented in Figure 1. The concentration of deprotonated sites is standardized per mass of dry biomass (mol/g) and calculated according to Fein et al. (1997). Titrated bacterial suspension exhibited a protonation–deprotonation behavior over the whole pH range studied. During the titration, no evidence of saturation was observed with respect to proton adsorption. The intersection point at zero charge appears between pH 6.48–6.77, being consistent with the experimental pH of zero proton charge (pH_zpc_) set by Protofit 2.1 rev1 (6.61 ± 0.07). The obtained pH*zpc* value indicated that Be9 strain exhibited a net negative charge at circumneutral pH which could bind positively charged metal species such as uranium and other heavy metals. Supplementary Table 2 summarizes the deprotonation constants and surface concentrations values of *Microbacterium* sp. Be9 and that of other bacterial strains for comparison purposes. The obtained pKa values are representative of carboxylic groups for pK_1_ (4.38), phosphate groups for pK_2_ (6.07) and amine and hydroxyl groups for pK_3_ (9.82). The results of potentiometric titration experiments showed that the cell surface functional groups with metal binding potential are carboxyl groups (pK_1_ around 4.3), phosphate groups (pK_2_ around 6), and hydroxyl and amine groups (pK_3_ around 9.8). These findings are in agreement with previous studies on bacterial surfaces (Haas et al., 2001; Merroun et al., 2011; Moll et al., 2014; Yu et al., 2014; Ruiz-Fresneda et al., 2020).

Surface site concentrations obtained were normalized to the dry mass of bacteria, resulting 0.45 ± 0.006 mol/g × 10^−4^ for acidic sites, 0.76 ± 0.015 mol/g × 10^−4^ for neutral sites and 1.19 ± 0.061 mol/g × 10^−4^ for basic sites. The surface of Be9 cells exhibit low concentrations of phosphates (0.76 ± 0.015 × 10^−4^ mol/g) compared to those of other bacterial species (Supplementary Table 2) such as *Stenotrophomonas bentonitica* (10.78 ± 0.31 × 10^−4^ mol/g; Ruiz-Fresneda et al., 2020), *Sporomusa* sp. MT-2.99 (5.30 ± 0.8 × 10^−4^ mol/g; Moll et al., 2014), *Sphingomonas* sp. S15–S1 (3.16 ± 0.56 × 10^−4^ mol/g; Merroun et al., 2011), or *Bacillus sphaericus* JG-7B (2.19 ± 0.25 × 10^−4^ mol/ g; Merroun et al., 2011). Similarly, the concentrations of carboxyl and hydroxyl/amine groups are also lower than those described above for other bacterial strains. However, strains such as *Shewanella putrefaciens* (Haas et al., 2001) or *Bacillus licheniformis* (Yu et al., 2014) have shown comparable values to Be9 strain.

#### 3.1.2. X-ray photoelectron spectroscopy

**Figure 2:**
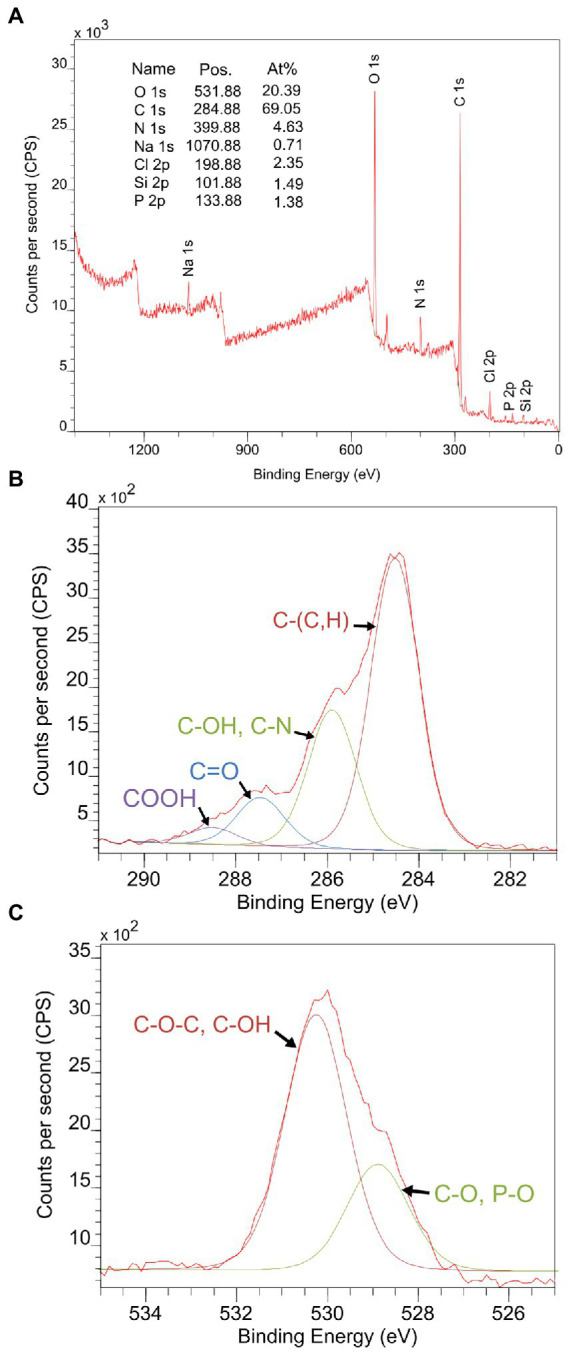
**(A)** X-ray photoelectron spectroscopy (XPS) survey spectrum collected from viable cells of *Microbacterium* sp. Be9 as a wide scan. The peaks of carbon **(B)** and oxygen **(C)** were scanned at high resolution and deconvoluted to assess the local chemical environment of these two elements.

In order to identify the elemental composition and estimate the concentration of main constituents of the *Microbacterium* sp. Be9 cell surface, XPS analysis was carried out using an X-ray photoelectron spectrometer (Kratos Axis Supra XPS). Binding energy curves of viable Be9 cells as a wide scan is shown in Figure 2A. A phosphorous peak appeared at a binding energy of 134.02 eV, and was attributed to phosphate groups (Ahimou et al., 2007; Ojeda et al., 2008), and the nitrogen peak at 400.02 eV attributable to amine or amide groups present in proteins (Ojeda et al., 2008; Kjærvik et al., 2018). Carbon and oxygen peaks were detected around 285 and 532 eV, respectively (Leone et al., 2006; Ojeda et al., 2008; Kjærvik et al., 2018) and analyzed at high resolution and deconvoluted to assess the contributions from each element (Figures 2B, C). The carbon peak was fit into four components: carbon bound to carbon or hydrogen [C–(C, H)], at 284.52 eV; carbon bound to nitrogen or hydroxyl group from amines, amides and/or alcohols [C–(OH, N)] at 285.89 eV; carbon doubly bound to oxygen from esters, acetals, carbonyls or carboxylates [C=O], at 287.46 eV; and carbon from carboxyl group [COOH], at 288.51 eV. The oxygen peak was decomposed into two components: oxygen double bound to carbon or phosphorous constituted in esters, carbonyls, amides, carboxylic acids, carboxylates or phosphoryl groups [C=O, P=O], at 528.89 eV; and oxygen present in phosphate, acetal, hemiacetal or hydroxide groups [P–OH, C–O–C and C–OH], at 530.27 eV.

The elemental concentration was estimated assuming polysaccharides, peptides and hydrocarbon-like products (such as lipids) as the main constituents on the bacterial surface. Based on previous studies (Rouxhet et al., 1994; Dufrêne et al., 1997; Van Der Mei et al., 2000), the molecular composition has been calculated by comparing the carbon concentration (in mmol/g) and the atomic concentration ratio of nitrogen and oxygen with respect to carbon for glucan (C_6_H_10_O_5_)_n_ for polysaccharides, the major outer membrane protein of *Pseudomonas fluorescens* OE 28.3 for peptides and using C–(C,H)/C = 1.000 from (CH_2_)_n_ for hydrocarbon-like products. Considering those compositions, a set of three equations can be provided:


(1) $$\frac{O}{C}=0.325\;\left(\frac{\text{CPEP}}{C}\right)+0.833\;\left(\frac{\text{CPS}}{C}\right)$$

(2) $$\frac{N}{C}=0.279\;\left(\frac{\text{CPEP}}{C}\right)$$

(3) $$1=\left(\frac{\text{CPEP}}{C}\right)+\left(\frac{\text{CPS}}{C}\right)+\left(\frac{\text{CHC}}{C}\right)$$

*Microbacterium* sp. Be9 proportions for each constituent were CPEP/C = 0.52, CPS/C = 0.75 and CHC/C = 0.69. Thus, the estimated concentrations of main constituents in the Be9 cell surface are 26.7% peptides, 38.2% polysaccharides and 35.1% hydrocarbon-like compounds.

### 3.2. U chemical speciation

The chemical speciation of 100 μM U in MOPS buffer [in the absence of G2P (MC1) and with the addition of G2P (MC2)] and in LPM (MC3) at pH 6.6 were determined using Visual MINTEQ 3.1 software (Gustafsson, 2020) and PhreeqC software (calculated using PRODATA thermodynamic database; Reiller and Descostes, 2020), respectively (Supplementary Table 3). In MC1/MC2 systems, the presence/absence of G2P showed no differences in the U speciation, dominated by hydroxo-uranyl complexes as (UO_2_)_3_(OH)^5+^ (78.3–78.5%) and (UO_2_)_4_(OH)^7+^ (19.5–19.2%). In contrast, U speciation in MC3 medium showed the presence of hydroxo-uranyl complexes [(UO_2_)_3_(OH)^5+^ (48.6%) and (UO_2_)_4_(OH)^7+^ (25.1%)] and U hydroxy-carbonates ((UO_2_)_2_(OH)_3_CO_3_^−^; 24.2%). In addition, U phosphate (UO_2_PO_4_^−^) was also identified (0.38%). In this case, a high positive saturation index was calculated, indicating a probable precipitation of inorganic uranyl orthophosphate (UO_2_)_3_(PO_4_)_2_·4H_2_O.

### 3.3. Effect of different phosphate species on Be9-U interactions

Our study aimed to investigate the effect of phosphates on the performance of the U biomineralization mediated by cells of *Microbacterium* sp. Be9. Three specific conditions using different phosphate sources were assayed (see above). A combination of wet chemistry (U removal kinetics, orthophosphate release monitoring), flow cytometry (cell viability assays), biochemical (phosphatase activity) and microscopic techniques were applied.

#### 3.3.1. Phosphate free system (MC1)

**Figure 3:**
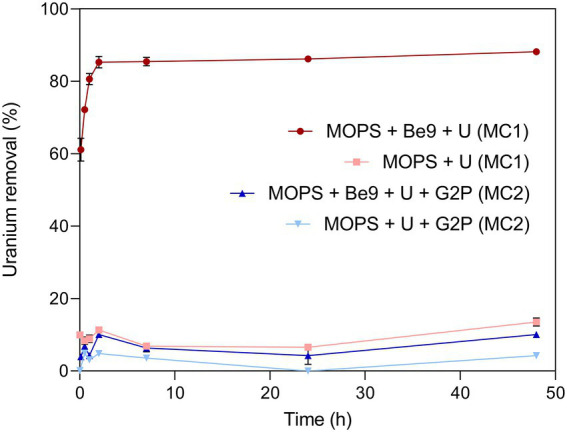
Uranium removal efficiency (%) kinetics during MC1 and MC2 conditions assayed. Abiotic treatments were used as control in both conditions. Data are shown as the mean and error bars represent standard error of three independent measurements.

Uranium removal kinetics by Be9 cells under absence of exogenous phosphates was determined by measuring residual U in the supernatants. The results indicated that the cells removed 60 and 88% of the initially added U within the first minutes and after 48 h of incubation, respectively (Figure 3). Abiotic controls consisting of U added to MOPS buffer showed that metal removal only reached up to 13% after 48 h of exposure, supporting the key biotic role in the high precipitation of U detected. To investigate whether phosphatases are involved in the U removal process, the activity of these enzymes and the associated orthophosphate released during the experiment were determined. The results showed no phosphatase activity in the U treated cells (Supplementary Figure 1). A very low release of inorganic phosphates was detected in this assay (2.21 mg/ml; Supplementary Figure 2A), coinciding with a non-significant phosphatase activity.

**Figure 4:**
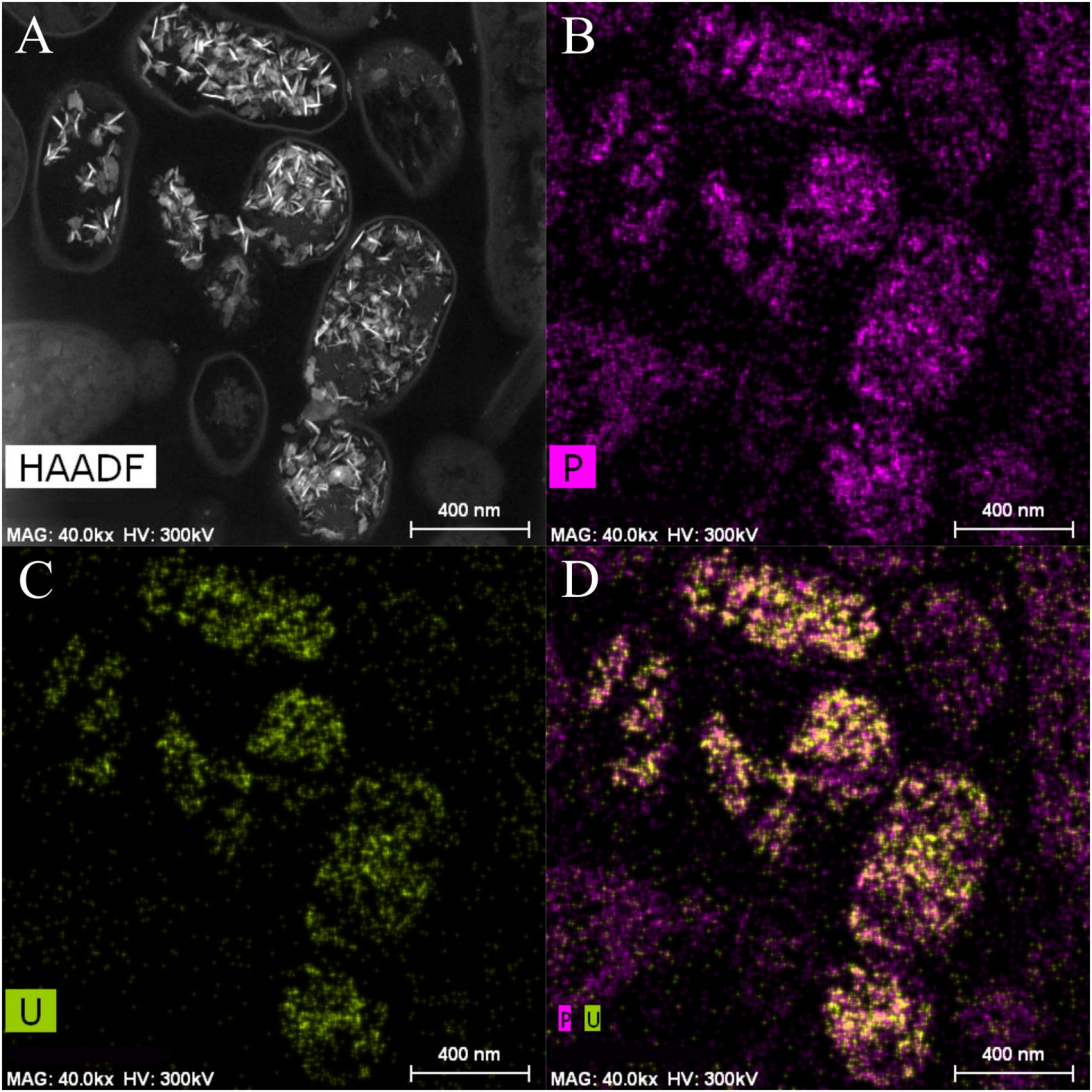
High angle annular dark-field-scanning transmission electron microscopy (HAADF-STEM) image of *Microbacterium* sp. Be9 cells after incubation under MC1 condition **(A)**; EDX element mapping for P **(B)**, U **(C)** and P–U combined **(D)**.

Scanning Transmission Electron Microscopy (STEM) micrographs of U-treated cells at 48 h showed that this radionuclide is mainly located intracellularly as needle-like fibrils (Supplementary Figure 3). The number of intracellular accumulates apparently increased according to the incubation time. No extracellular U precipitates were observed. Elemental mapping analysis illustrated in Figure 4 shows that the U precipitates were composed of U and P, probably revealing a biomineralization of the U phosphates. We used flow cytometry technique based on live/dead staining to investigate the effect of U in the cell viability and cell membrane potential. 100% of untreated (control treatment) and U-treated Be9 cell populations remained viable and metabolically active for the first 24 h. The results suggest that the intracellular accumulation of U at 24 h (86% of accumulated U) and the consequent biomineralization of U phosphates is a metabolically active process associated with biological activity of the strain Be9. However, at 48 h, the viability of the U-treated cells decreased to 18% and the membrane potential of the cells was markedly reduced to 0% (Supplementary Table 4).

#### 3.3.2. System supplemented with organic phosphates (MC2)

**Figure 5:**
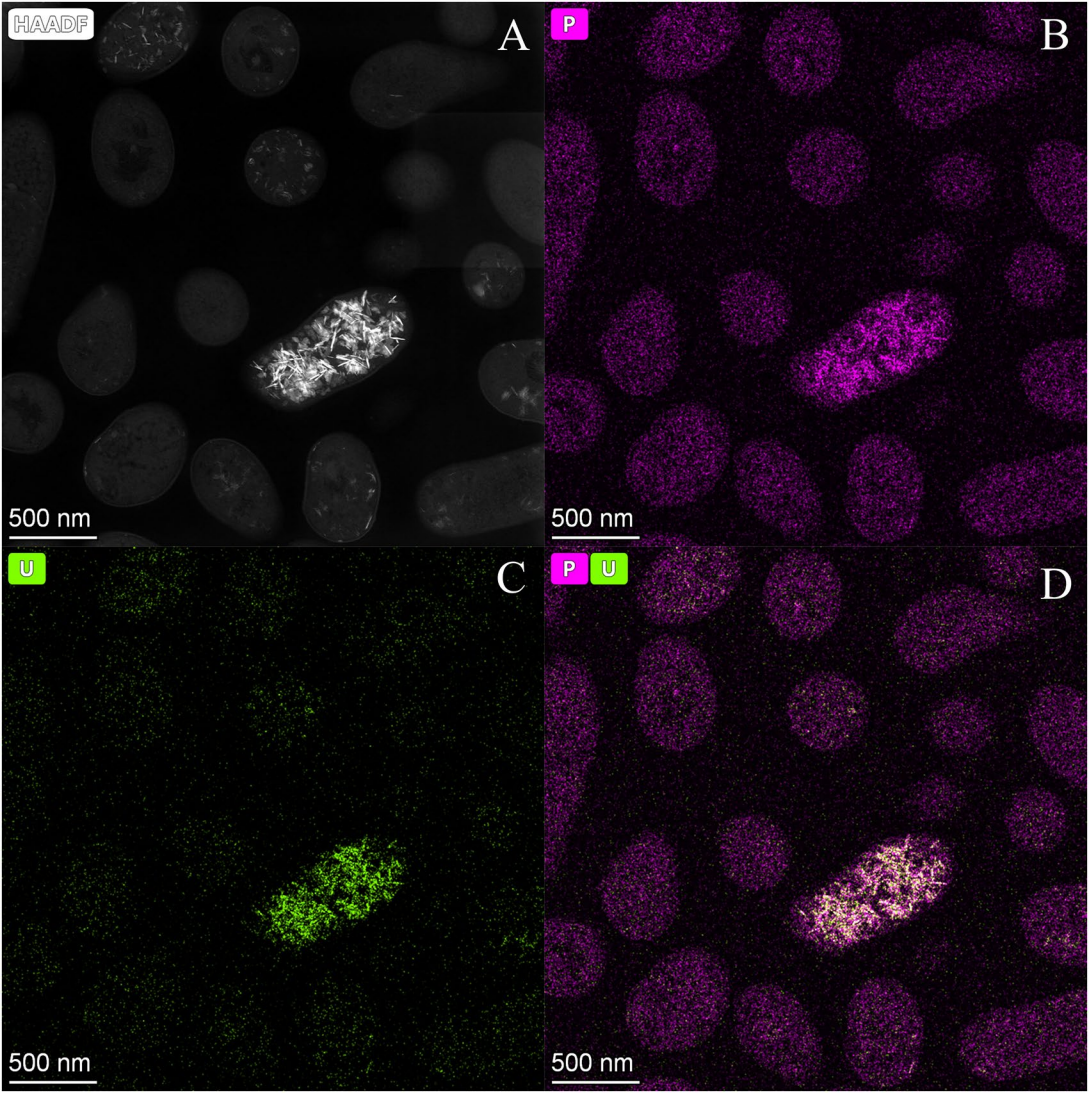
High angle annular dark-field-scanning transmission electron microscopy (HAADF-STEM) image of *Microbacterium* sp. Be9 cells after incubation under MC2 condition **(A)**; EDX element mapping for P **(B)**, U **(C)** and P–U combined **(D)**.

We investigated the effect of an exogenous organic phosphate substrate (G2P) in the U biomineralization. When G2P was added, U-removal by Be9 cells was about 10% after 48 h (Figure 3), significantly lower than that in phosphate-free system (MC1 system; see above). In the abiotic control treatments, consisting of the MOPS buffer treated with G2P and U, the removal rate was only 4.3% of the initial U concentration. At the end of the experiment, the results indicated that, in the presence of organic phosphates, bacterial cells play a minor role in U removal. Despite the addition of G2P, low levels of Pi were released, in accordance with the weak acid phosphatase displayed (Supplementary Figure 2A). Considering that the added G2P concentration (5 mM) was equivalent to 475 mg/l of Pi, the amount of Pi released to the supernatant was low (10.59 mg/l), similar to the MC1 condition shown above (Supplementary Figure 2A). In the presence of G2P an increase inBe9 cell viability was measured from 24 h (61.03% viable cells) to 48 h (85.14% viable cells), but their cell membrane potential at 48 h was reduced in comparison with the non-G2P treatment (Supplementary Table 4). STEM micrographs of thin sections of U-treated cells in the presence of G2P showed a few U intracellular accumulates (Supplementary Figures 4, 5). These U accumulates appeared as needle fibrils as well as immobilized within the granules of the phosphates. The detection of a low number of U accumulates are in line with the low levels of U removal (10%). Elemental mapping analysis of certain Be9 cells that showed precipitation revealed that U appeared co-localized with P (Figure 5), as in the phosphate-free system (MC1).

#### 3.3.3. Effect of inorganic phosphates in U biomineralization (MC3)

**Figure 6:**
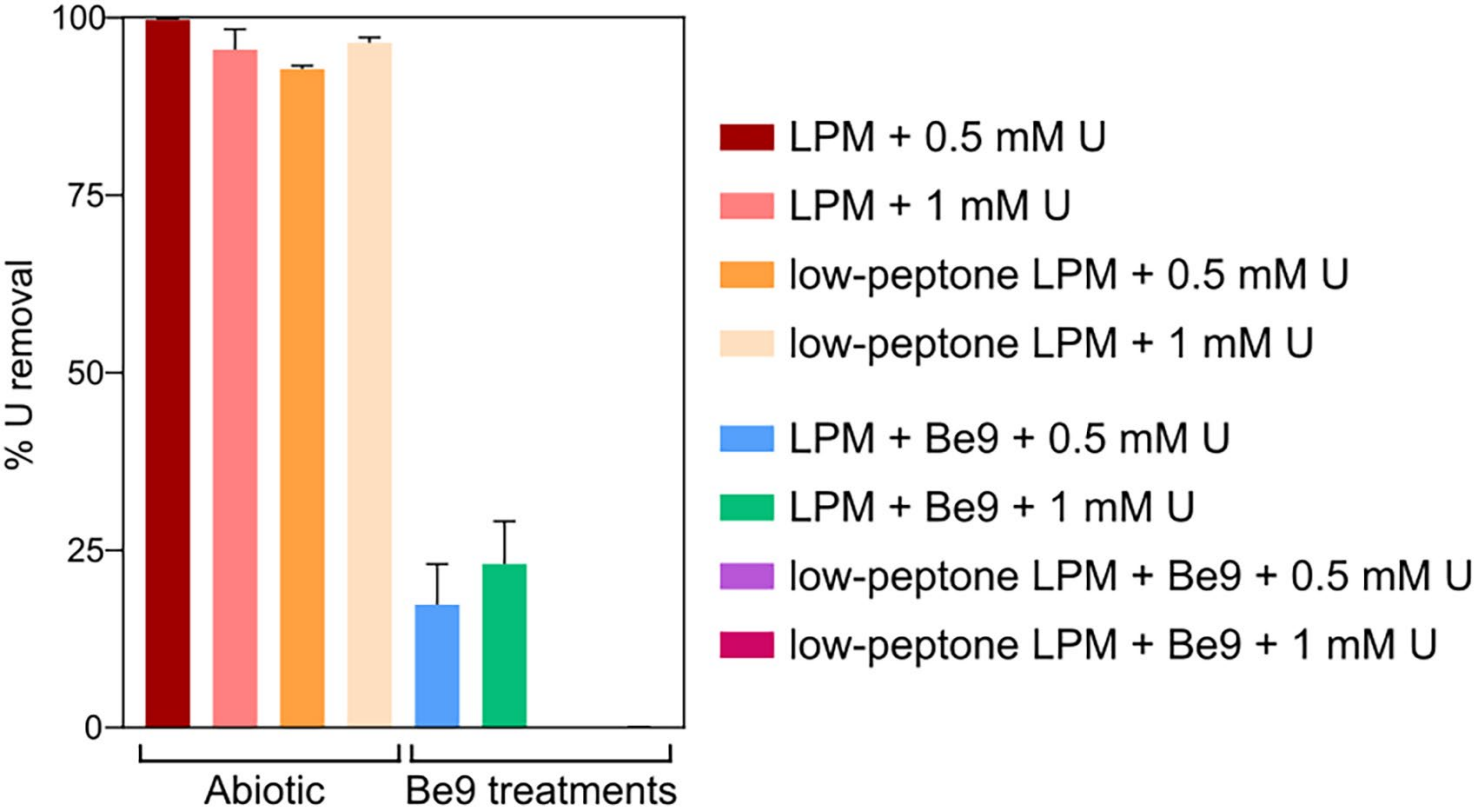
Uranium removal after the incubation under different phosphate and U concentrations in the presence and absence of *Microbacterium* sp. Be9 cells. Peptone reduction (0.2 mg/l) is labeled as low-peptone low phosphate medium (LPM). Data are shown as the mean and the error bars represent the standard error of at least three independent measurements.

**Figure 7:**
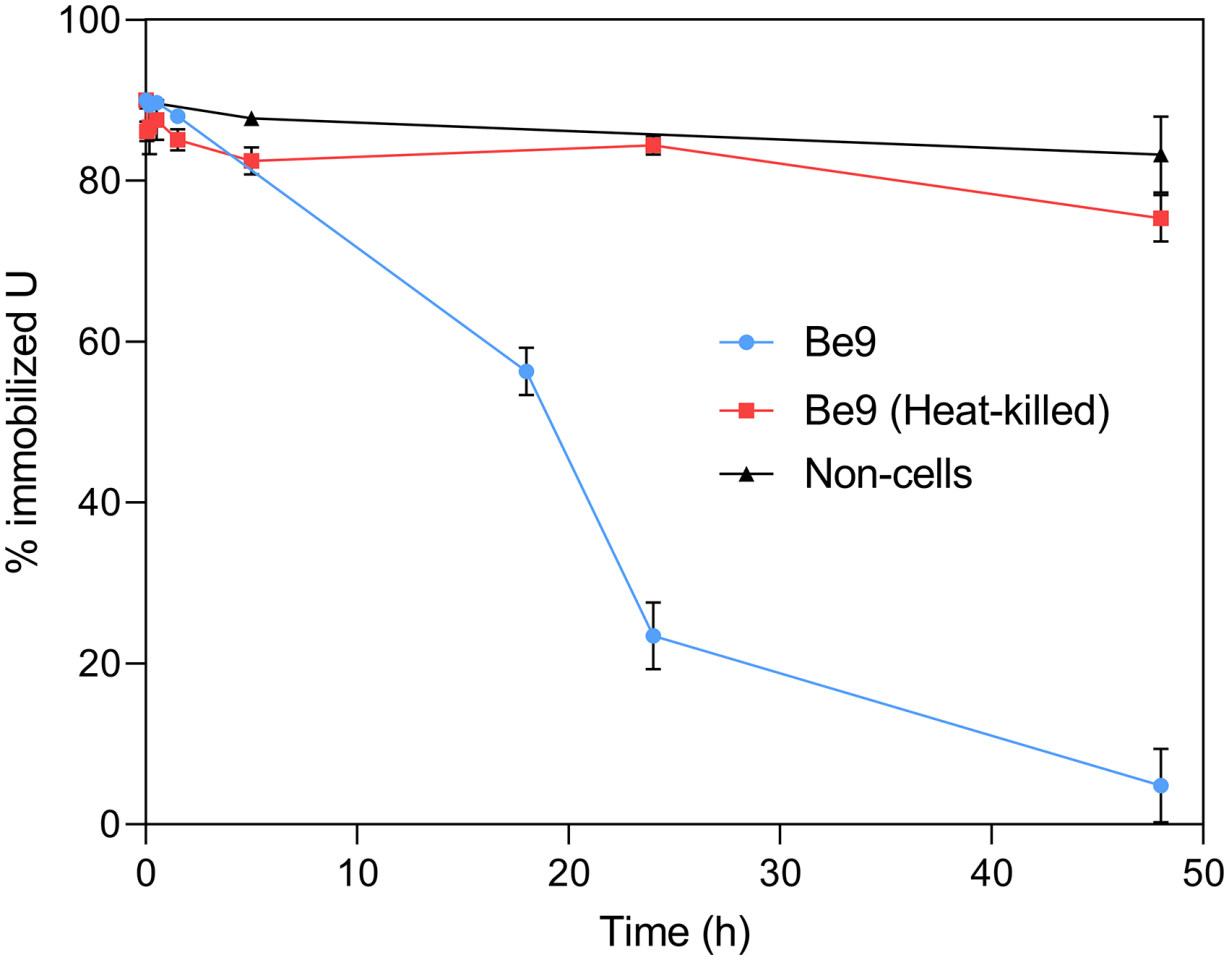
Uranium solubilization kinetics assay (%) during the incubation of *Microbacterium* sp. Be9 cells with U-phosphate precipitates. Flasks without cells and including heat-killed bacterial cells were used as control treatments. Data are shown as the mean and the error bars represent the standard error of at least three independent measurements.

We studied the effect of inorganic phosphates in the U interaction by the strain Be9 using low phosphate medium (LPM) at two U concentrations (0.5 and 1 mM). Peptone was used as source of phosphate at different concentrations (100 and 0.2 mg/l) in order to consider the theoretical abiotic precipitation of U according to the speciation analysis previously presented (Supplementary Table 3). Under abiotic conditions, almost all the U was removed from the solution (93–99%; Figure 6), supporting the precipitation of the metal as indicated by U chemical speciation studies. However, in the treatments with Be9 strain and LPM supplemented with U, the elimination was 13 and 23% of U at 0.5 and 1 mM metal concentration, respectively, after 48 h (Figure 7). Moreover, when the Be9 cells were inoculated in low-peptone LPM (0.2 mg/l) supplemented with 0.5 and 1 mM U, non-metal removal was observed, with 100% of the U remaining in the soluble form.

**Figure 8:**
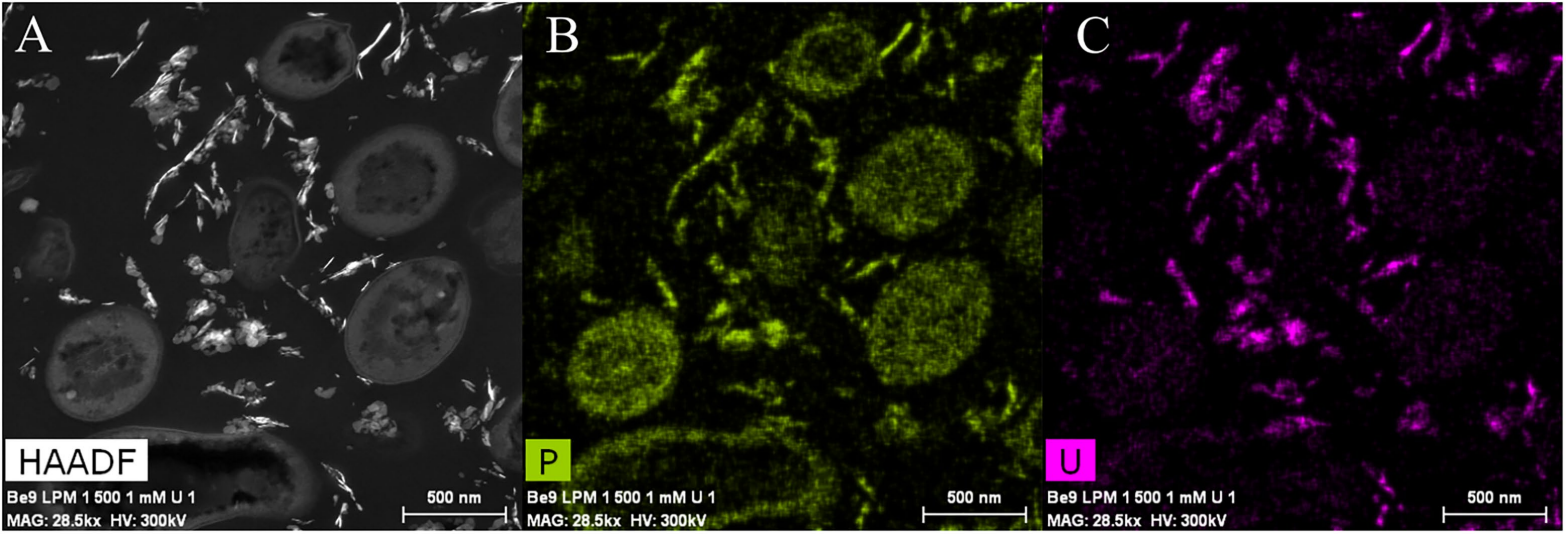
High angle annular dark-field-scanning transmission electron microscopy (HAADF-STEM) image of *Microbacterium* sp. Be9 cells after incubation under MC3 treatment (0.2 mg/l peptone concentration and 1 mM U) **(A)**; EDX element mapping for P **(B)** and U **(C)**.

Phosphatase activity of the Be9 strain was only detected after incubation in the LPM medium under 0.5 and 1 mM U concentrations (Supplementary Figure 1), whilst the low-peptone condition assays exhibited no enzyme activity. Measurements of the released Pi (Supplementary Figure 2B) revealed that the cells were able to solubilize 20 and 25 mg/l of orthophosphates from the solution supplemented with 0.5 and 1 mM U, respectively. Under peptone-reduced treatments with 0.5 and 1 mM U concentrations, the Be9 strain also released similar amounts to before (27 and 24 mg/l, respectively). The Pi concentration detected after incubation in abiotic control treatments was around 0.02–3 mg/l due to its interaction with U, except at 0.5 mM U concentration, which was 47.40 mg/l. Be9 cell viability and activity in the LPM medium remained over 98% for 48 h under phosphate reduction and both U concentrations tested (Supplementary Table 4). STEM-HAADF micrographs of U precipitates formed abiotically in LPM (100 and 0.2 mg/l peptone concentration) supplemented with 0.5 and 1 mM U for 48 h revealed the presence of solid phases with different sizes and morphology (aggregates, needle-like fibrils, etc.; Supplementary Figure 5). In the presence of the Be9 strain, STEM micrographs did not show any U precipitates (Supplementary Figure 6), according to the U removal levels measured before. Only some U precipitates were detected in the extracellular space which could correspond to abiotic U precipitates. Elemental mapping analysis showed that these extracellular precipitates were mainly composed of U and P indicating the precipitation of the abiotic U phosphates (Figure 8).

#### 3.3.4. Uranium solubilization kinetic assay

It is well known that *Microbacterium* species from diverse natural environments are classified within the group of PSB (Panda et al., 2016; Zhang et al., 2017). Therefore, we further studied the uranium solubilization process detected through a kinetic assay using living and heat-killed Be9 cells. Abiotic U-phosphate precipitates recovered from LPM supplemented with 0.5 mM U for 48 h (as detected previously), were inoculated with active and heat-killed cells of Be9 strain. In addition, LPM supplemented with 0.5 mM U was also considered as an abiotic control. In the presence of active cells, the solubilization of U phosphates increased gradually throughout the incubation time, reaching around 25% of U immobilized at 24 h and 5% at 48 h (Figure 7). In heat-killed cells and abiotic control treatments, 90% of the U amended phosphates remained intact within the 48 h of exposure. These results indicate that the biological activity of the strain *Microbacterium* sp. Be9 was directly involved in the U solubilization.

## 4. Discussion

The main objective of this study is to investigate the effect of phosphates in the biomineralization of U (VI) by *Microbacterium* sp. Be9, a uranium mill tailings porewater isolate. To the best of our knowledge, this the first study determining the effect of organic and inorganic phosphates in the U phosphate biomineralization process. Previously, environmental factors such as pH (Beazley et al., 2007; Chandwadkar et al., 2018; Zhang et al., 2018), temperature (Li et al., 2013), the presence of carbonates (Wei et al., 2019), NH_4_^+^ incorporation (Yong and Macaskie, 1995) and organic matter (Medina et al., 2017; Boiteau et al., 2018) have been described to affect the U phosphate biomineralization. Our experiments have shown that the U biomineralization ability by the Be9 strain is drastically reduced by both organic and inorganic phosphates. However, in an exogenous phosphate-free system, the cells removed up to 88% U within 24 h (Figure 3) through intracellular U phosphate biomineralization. In addition, as a result of the kinetic studies, we can confirm that in the presence of previously abiotically precipitated U phosphates, the Be9 cells were able to solubilize this radionuclide (Figure 7) through a metabolism-dependent process.

The first step in this microbial U biomineralization process by the Be9 strain appears to be surface biosorption. Bacterial surfaces act as nucleation sites for the formation of biogenic U(VI)-phosphates through combined surface sorption of this radionuclide and phosphatase-mediated U biomineralization (Pan et al., 2015; Theodorakopoulos et al., 2015). Membranes play a major role in sorption of metals where electrostatic interactions between positively charged metal species and negatively charged functional groups (e.g., phosphates and carboxyles) occur. Gram-positive bacteria (such as the Be9 strain) exhibit greater biosorption capacity than Gram-negative bacteria due to their cell envelope components (Hufton et al., 2021). Liu et al. (2015) demonstrated the role of carboxyl, phosphoryl, and amino functional groups of *Synechococcus* sp. PCC 7002 cells as metal surface ligands by means of potentiometric titrations. Therefore, in our study, the chemical properties of the cell surface of Be9 strain were characterized using potentiometric titration and XPS analysis. The pH zero proton charge (pH_zpc_) around 6.61 ± 0.07 (Supplementary Table 2) indicated that Be9 cells developed a negative net charge at the pH value we studied. These data show that electrostatic attraction of negatively ionizing groups (e.g., carboxyl, phosphoryl and hydroxyl groups) with positively charged U species [e.g., (UO_2_)_3_(OH)^5+^ and (UO_2_)_4_(OH)^7+^] is favorable. Thermodynamic calculation showed that under phosphate free system and in presence of G2P, these favorable compounds as (UO_2_)_3_(OH)^5+^ and (UO_2_)_4_(OH)^7+^ represented 78 and 20% species of U (Supplementary Table 3). In the case of phosphate-free system, the negatively ionizing groups of the cell wall could sorb 70% of positively charged U species in the first 10 min as was shown by the U removal kinetics studies (Figure 3). XPS analysis exhibited a high proportion of polysaccharides in the Be9 cell surface. Polysaccharides have been described as playing an important role in enhancing the tolerance of microorganisms under metal stress (Sun et al., 2020). In the cell surfaces of *Pseudomonas putida* similar percentages of peptides were obtained and were involved in the interaction with nZVI/Pd particles (Lv et al., 2017).

Under the absence of phosphates (MC1 system), a metabolism dependent precipitation of U intracellular phosphates seemed to take place after the surface sorption. The U removal capacity increased slowly throughout the time, without any significant differences between 24 and 48 h. These results show that U removal by this strain is a biphasic process with a fast first phase mediated by a passive process (e.g., sorption at cell surfaces) and a slow second phase driven by metabolically active processes (e.g., intracellular accumulation, biomineralization, etc.). However, in comparison to other bacterial strains, Be9 showed low concentrations of phosphate or carboxyl groups at the cell surface (Supplementary Table 2) which could probably explain why U is mainly accumulated intracellularly as was demonstrated by electron microscope analysis. Previous studies showed a strong correlation between the concentration of phosphates and carboxyl groups of the cell wall and cellular localization of the metal of some bacterial strains such *S. bentonitica* BII-R7 (Sánchez-Castro et al., 2020). The latter strain exhibited 12 and 10 orders of magnitude of phosphate and carboxyl groups (Ruiz-Fresneda et al., 2020) higher than the Be9 strain, and high capability to bind and biomineralize U at the cell surface (Sánchez-Castro et al., 2020). However, in spite of the low amount of phosphate and carboxyl groups, seems to be sufficient to act as a first passive sorption step followed by intracellular precipitation of uranium.

Biphasic U removal process has also been reported by other authors in different bacteria such as *Acidovorax facilis* (Gerber et al., 2016)*, Stenotrophomonas bentonitica* BII-R7 (Pinel-Cabello et al., 2021) and *Microbacterium* sp. A9 (Theodorakopoulos et al., 2015). Flow cytometry studies revealed that 100% of the cells were viable and metabolically active during the first 24 h, supporting the involvement of a metabolic-dependent process in the interaction with U besides biosorption. A decrease in cell viability at 48 h seems to be clearly related to the high U-bioprecipitation inside the cells. In the case of uranium, numerous studies have reported the immobilization of this radionuclide by intracellular polyphosphate bodies through different analyses as TEM and EDX (Merroun et al., 2003, 2006; Suzuki and Banfield, 2004; Li et al., 2016). The low concentrations of phosphate and carboxyl groups at the Be9 cell surface indicated by potentiometric titration studies (Supplementary Table 2) could explain the low U amount bound to the surface. However, in spite of the low amount of phosphate and carboxyl groups, seems to be sufficient to act as a first passive sorption. In our study, the presence of U in the cells is speculated to induce the activity of enzymes such as exopolyphosphatase to degrade polyphosphate, thus releasing orthophosphates for the biomineralization of U. Theodorakopoulos et al. (2015) proposed an induction of enzymatic pathways from a *Microbacterium* strain which causes bound breakage of polyphosphate granules in the cytoplasm for the biomineralization of U intracellularly. However, the intracellular biomineralization of U associated with degradation of polyphosphates require the U uptake by the cells. For metal uptake in microorganisms, two main mechanisms were described: first, an active process through metal transporters for essential metals, and then, a passive process by an increase of cell membrane permeability due to damage for toxic metals (Suzuki and Banfield, 1999). In the case of *Microbacterium*, it has been demonstrated that iron transportation proteins are involved in the uranium uptake by *Microbacterium oleivorans* A9 (Gallois et al., 2018). A recent study about different *Microbacterium* strains revealed a transmembrane protein with binding affinity for UO_2_^2+^ and Fe^3+^ specific to U-tolerant strains (Gallois et al., 2022). Regarding to the Be9 strain, in a previous study, its genome was annotated and ABC-type Fe^3+^ siderophore transport proteins, which may be involved in U uptake, were identified (Martínez-Rodríguez et al., 2020). Transcriptomics and proteomics studies are needed to further address the nature of transporters used for U intracellular accumulation in the strain Be9.

Scanning transmission electron microscopy (STEM) micrographs of Be9 cells showed a gradual formation of needle-like structures composed of U and P in the cytoplasm over time. The U phosphate nature of similar needle-like structures was confirmed in the literature by the use of different spectroscopic techniques such as EXAFS, TRLFS, etc. (Lopez-Fernandez et al., 2018), also in *Microbacterium* genus (Theodorakopoulos et al., 2015). Microbes are able to precipitate U phosphate phases with different structures (e.g., autunite, chernikovite, etc.) through phosphatase activity which degrade organic phosphates (e.g., glycerol phosphates) and release orthophosphates (Merroun et al., 2011; Skouri-Panet et al., 2018; Sánchez-Castro et al., 2020). In our study, although phosphatase activity was not detected, U phosphates were evidenced in the cells treated with U. Thus, the U phosphate biomineralization induced by this strain is probably not associated with phosphatase activity. Similarly, other U-tolerant *Microbacterium* strains showed a very low abundance of acid and alkaline phosphatase activity in their proteome (Gallois et al., 2022). The needle-like structures identified in STEM micrographs could be raised from the interaction of U species with existent orthophosphates in intracellular polyphosphates since no exogenous phosphate substrate was added. Previous studies have reported some bacterial strains capable of precipitating U into uramphite without additional phosphorous sources (Pan et al., 2015; Zhang et al., 2018). Polyphosphates (polyPs) granules are ubiquitous polymers occurring in many microorganisms (Seufferheld et al., 2008). They contain a high number of orthophosphate residues that are linked to phosphoanhydride bonds to form linear polymers (Mandala et al., 2020). However, a recent study described the existence of small metaphosphates, cyclic oligomers of [PO_3_]^(−)^ made up of 3 to 8 phosphate groups (Mandala et al., 2020) as a candidate for precipitation of positively charged U species. Polyphosphate bodies possess various biological functions including phosphate removal, metal chelation, stress response, etc. (Achbergerová and Nahálka, 2011). Previous studies have demonstrated the role of bacterial polyphosphates in the accumulation of heavy metals and radionuclides (Brim et al., 2000; Acharya and Apte, 2013; Villagrasa et al., 2020). Be9 whole genome sequence analysis indicated the presence of genes codifying for enzymes involved in the synthesis (e.g., polyphosphate kinase) and degradation (e.g., exopolyphosphatase) of polyphosphates (Martínez-Rodríguez et al., 2020). In addition, *Microbacterium* spp. are well documented as potential polyphosphate accumulating microorganisms (Theodorakopoulos et al., 2015; Suzina et al., 2022). Therefore, the results obtained are in agreement with the expected hypothesis, where Be9 cells are able to release orthophosphates for the U biomineralization through polyphosphatase activity on intracellular polyphosphate granules. No phosphatase activity was involved in this metal/bacteria interaction process.

Surprisingly, in MC2 system (G2P presence), Be9 cells exhibited low U removal capacity contrary to expected, as compared to free phosphate system. In addition, no phosphatase activity was detected. Microbial phosphatase enzymes (acid and alkaline) catalyze the hydrolysis of G2P into glycerol and orthophosphates which lead to the precipitation of U phosphates with different structures such as efficient strategy for U bioremediation (Beazley et al., 2011; Merroun et al., 2011; Liang et al., 2016; Sánchez-Castro et al., 2020, 2021). However, although no phosphatase activity was measured, a certain concentration of orthophosphates was detected under these conditions. Thus, since G2P is not degraded extracellularly, it is probably transported intracellularly to be used as an organic carbon source for the metabolism of carbohydrates and lipids. This hypothesis is supported by the fact that the whole genome sequence of Be9 exhibited genes codifying for proteins for the uptake and utilization of G3P and glycerol (glycerol-3-phosphate ABC transporter, ATP-binding protein UgpC, glycerol-3-phosphate dehydrogenase, glycerophosphoryl diester phosphodiesterase). Gallois et al. (2018) reported the upregulation of proteins involving transport of glycerol-3-phosphate in the cells of *Microbacterium oleivorans* A9 treated with uranium. In contrast with the previous conditions (phosphate-free system), cell viability under the presence of U and G2P increased from 24 to 48 h of incubation, which supports the assumption that G2P is used by Be9 cells as a carbon or phosphorus source. Tu et al. (2019) described that strong organic-ligands such as oxalate and citrate competed with biotic PO_4_^3−^ for uranyl, inhibiting the U-phosphate biomineralization by the cells of *Bacillus* sp. dw-2. Speciation of U in MC2 system (Supplementary Table 3) was dominated by hydroxo-uranyl complexes as (UO_2_)_3_(OH)^5+^ (78.51%) and (UO_2_)_4_(OH)^7+^ (19.22%) and no G2P-U complexes were expected. However, the mechanisms responsible for the decrease of U removal and biomineralization rate remains unclear.

Finally, we used the culture medium LPM in different treatments (MC3 system) to induce abiotic precipitation of U phosphates. As expected, the chemical speciation of U (at 0.5 and 1 mM) in this medium and in its diluted form (0.2 mg/l peptone concentration) resulted in a high positive saturation index (Supplementary Table 3), predicting the precipitation of various uranyl compounds including U phosphates. These results were confirmed by abiotic precipitation experiments where U precipitation reached high values after 48 h (Figure 6). Numerous amorphous structures such as U-phosphates and U-carbonates were identified in HR-TEM micrographs by EDX analysis (Supplementary Figure 7). However, in presence of Be9 cells, 94.5% of U was re-solubilized from abiotically precipitated U phosphates. Under this U concentration, the Be9 cells remained viable until 48 h, reacting with the precipitated U species, mainly U inorganic phosphates. Thus, contrary to the expected hypothesis, the obtained results demonstrated the *Microbacterium* sp. strain Be9 capacity of U phosphate solubilization, due to its belonging to PSB group. The cells seemed to display active mechanisms to solubilize this radionuclide. Microorganisms known as PSBs are able to solubilize both organic and inorganic phosphorus from insoluble compounds. Recently, numerous U-tolerant PSB strains have been isolated from mining sites of this radionuclide (Sowmya et al., 2014, 2020; Lv et al., 2022). Mechanisms of phosphate solubilization by PSB strains involve hydroxyl and carboxyl groups present in the cells or in released organic acids (Chen et al., 2006). In addition, a number of genes encoding enzymes responsible for P solubilization (e.g., encoding quinoprotein glucose dehydrogenase (*gcd*), phnP C-P lyase subunit (*phnP*), phoA alkaline phosphatase (*phoA*), phoD alkaline phosphatase (*phoD*), and phoN acid phosphatase (*phoN*)) have been reported (Rawat et al., 2021). In our study, we detected the U-mobilization activity by the Be9 strain reacting with inorganic phosphates. This fact indicates that this behavior may involve the solubilization of U from precipitated U-phosphates. In this way, bonded heavy metals (such as U) could be released in a soluble and toxic form to the environment. However, some bioremediation strategies require interaction with an available form of the metal to facilitate its removal. To this aim, the Be9 solubilization behavior could be applied in combined U remediation technologies.

## 5. Conclusion

In this study we have demonstrated that the *Microbacterium* sp. strain Be9 possesses a dual behavior toward U and is capable of precipitating and solubilizing this radionuclide under different conditions. In addition, the presence or absence of different phosphate sources had an influence on the U biomineralization ability of the Be9 strain. Therefore, this strain could be used as U bioremediation agent in a free exogenous phosphate system. And due to its potential in the solubilization of phosphates from organic and inorganic P sources, this strain could contribute indirectly to providing this inorganic anion for the precipitation of this radionuclide. Thus, our results provide new insights into the impact of microbes on the biogeochemical cycle of U in the presence of different forms of phosphates. This information leads to the understanding of the conditions regarding biogenic U (VI) phosphate mineral formation and the physico-chemical parameters which hinder biomineralization and remediation strategies in oxidizing conditions. The correct selection of the supplemented organic phosphate donor could be crucial depending on the microorganism used. Therefore, the importance of gaining knowledge about microbial diversity and its role with the target metal is crucial to achieve the correct application of selected bioremediation technologies.

## Data availability statement

The original contributions presented in the study are included in the article/Supplementary material, further inquiries can be directed to the corresponding author.

## Author contributions

PM-R, IS-C, and MM designed the experimental setup and methodology, and major contributors in writing the manuscript. PM-R and IS-C performed all laboratory works, analyzed the data, and created the graphs and figures. MA carried out microscopy analyses presented here. JO designed and supervised the cell surface analysis reported here. JO and MD revised the manuscript. MD and MM conceived the concept, led this project and obtained funds for carrying out these studies. All authors contributed to the article and approved the submitted version.

## Funding

This work was supported by ORANO Mining (France; collaborative research contract no 3022 OTRI-UGR). It results from a Joint Research Project between Orano Mining R&D Department and the Department of Microbiology of the University of Granada.

## Conflict of interest

The authors declare that the research was conducted in the absence of any commercial or financial relationships that could be construed as a potential conflict of interest.

## Publisher’s note

All claims expressed in this article are solely those of the authors and do not necessarily represent those of their affiliated organizations, or those of the publisher, the editors and the reviewers. Any product that may be evaluated in this article, or claim that may be made by its manufacturer, is not guaranteed or endorsed by the publisher.

## Footnotes

^1^ http://www.casaxps.com/

^2^ https://vminteq.lwr.kth.se/
