# Supplementary material for: Effect of different phosphate sources on uranium biomineralization by the Microbacterium sp. Be9 strain: A multidisciplinary approach study
Source: Front Microbiol. 2023 Jan 9;13:1092184. doi: 10.3389/fmicb.2022.1092184 (PMC9868770; doi:10.3389/fmicb.2022.1092184)
Supplement: Supplementary file 1 [file Data_Sheet_1.docx]

Supplementary Material

**Table Supplementary 1.** Low Phosphate Medium composition.

| *Saline solution 10×, pH 7.2 | 100 ml/L | |
| --- | --- | --- |
|  |  | |
|  | *Saline solution10X, pH 7.2, composition | |
|  | NaCl | 46.8 g/L |
|  | KCl | 14.9 g/L |
|  | NH_4_Cl | 10.7 g/L |
|  | (NH_4_)_2_SO_4_ | 4.3 g/L |
|  | MgCl_2_ | 10 ml/L |
|  | ZnSO_4_ | 2.7 mg/L |
|  | Tris-base | 143.3 g/L |
|  |  | |
| Glycerol | 5 ml/L | |
| Thiamine (50 mg/ml) | 0.4 ml/L | |
| Peptone 10% | 100 ml/L | |
| CaCl_2_ (100 mM) | 1 ml/L | |
| Destillated water | 793.6 ml/L | |

**Table Supplementary 2.** Comparison of deprotonation constants and surface site concentrations between *Microbacterium* Be9 and other six bacterial species from different studies.

| **Species** | **pK1** | **pK2** | **pK3** | **C_1_ (×10^-4^ mol/g)** | **C_2_ (×10^-4^ mol/g)** | **C_3_ (×10^-4^ mol/g)** | **pH_zpc_** | **Reference** |
| --- | --- | --- | --- | --- | --- | --- | --- | --- |
| *Microbacterium* sp. strain Be9 | 4.38 ± 0.67 | 6.07 ± 0.37 | 9.82 ± 0.12 | 0.45 ± 0.006 | 0.76 ± 0.015 | 1.19 ± 0.061 | 6.61 ± 0.07 | This study |
| *Stenotrophomonas bentonitica* | 4.97 ± 0.08 | 6.88 ± 0.02 | 9.43± 0.02 | 5.05 ± 0.31 | 10.78 ± 0.31 | 16.93 ± 1.45 | 5.7 | (Ruiz-fresneda et al., 2020) |
| *Sporomusa* sp. MT - 2.99 | 4.8 ± 0.06 | 6.68 ± 0.06 | 9.01 ± 0.08 | 5.3 ± 0.8 | 3.5 ±0.3 | 4.8 ± 0.5 | - | (Moll et al., 2014) |
| *Bacillus licheniformis* | 3.7 ± 0.2 | 5.5 ± 0.3 | 9.4 ± 0.3 | 0.59 ± 0.3 | 0.34 ± 4.9 | 0.50 ± 11.0 | - | (Yu et al., 2014) |
| *Shewanella putrefaciens* | 5.16 ± 0.04 | 7.22 ± 0.15 | 10.04 ±  0.67 | 0.32 ± 0.02 | 0.09 ± 0.01 | 0.38 ± 0.01 | - | (Haas et al., 2001) |
| *Sphingomonas* sp.  S15-S1 | 4.27 ± 0.45 | 7.03 ± 0.86 | 9.92 ± 0.32 | 4.91 ± 1.04 | 3.16 ± 0.56 | 9.24 ± 2.97 | 5.8 | (Merroun et al., 2011) |
| *Bacillus sphaericus* JG-7B | 4.37 ± 0.27 | 6.37 ± 0.31 | 9.95 ± 0.16 | 4.70 ± 0.55 | 2.19 ± 0.25 | 4.56 ± 0.77 | 5.5 | (Merroun et al., 2011) |

**Table Supplementary 3.** U speciation (0.1 mM) in MC1 (MOPS+U), MC2 (MOPS+U+G2P) and MC3 (LPM + U) treatments, as predicted by Visual MINTEQ software 3.1 and PhreeqC software. ^a^ aq, aqueous.

| U Species | % of total concentration | | |
| --- | --- | --- | --- |
|  | MC1 | MC2 | MC3 |
| UO_2_^2+^ | 0.05 | 0.06 | 0.02 |
| UO_2_OH^+^ | 1.10 | 1.14 | 0.64 |
| (UO_2_)_2_(OH)_2_^2+^ | 0.17 | 0.20 | 0.09 |
| (UO_2_)_3_(OH)^5+^ | 78.31 | 78.51 | 48.59 |
| (UO_2_)_2_(OH)_3_CO^3-^ | - | - | 24.25 |
| (UO_2_)_4_(OH)^7+^ | 19.52 | 19.22 | 25.14 |
| UO_2_PO^4-^ | - | - | 0.38 |
| UO_2_HPO_4_ | - | - | 0.07 |
| UO_2_CO_3_ | - | - | 0.04 |
| (UO_2_)_3_(OH)^7-^ | 0.05 | 0.05 | 0.12 |
| (UO_2_)_3_(OH)_4_^2+^ | 0.10 | 0.12 | 0.05 |
| UO_2_(OH)^3-^ | 0.03 | 0.03 | 0.06 |
| UO_2_(OH)_2_ (aq)*^a^* | 0.68 | 0.67 | 0.55 |


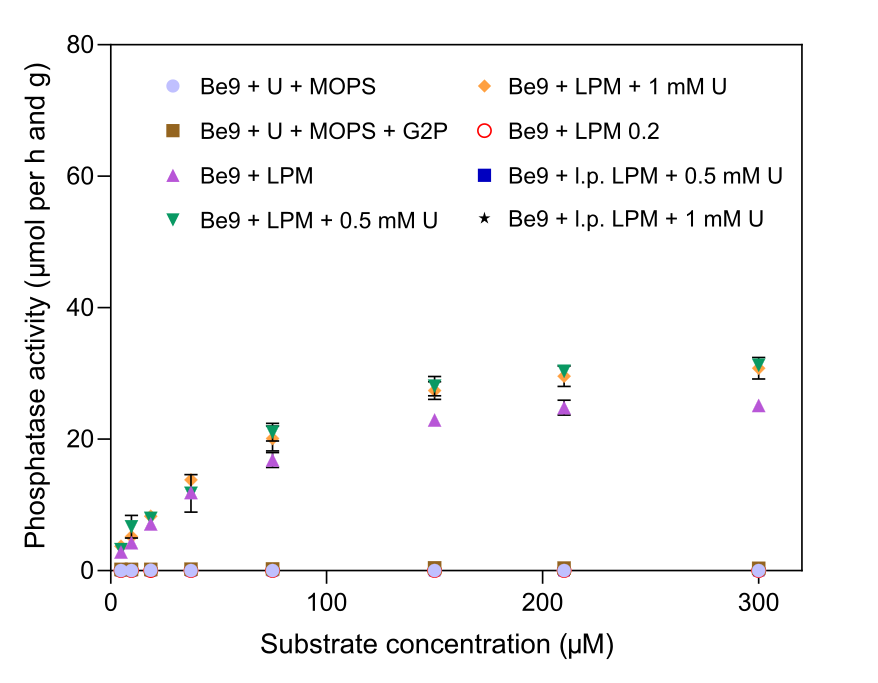


**Figure Supplementary 1.** Phosphatase activity (measured as µmol released per h and g at different substrate concentrations) of Be9 cells after incubation in the different conditions (MC1, MC2 and MC3). Peptone reduction (0.2 mg/L) is labelled as l.p. LPM (low-peptone LPM). Data are showed as the mean and error bars represent the standard error of at least three independent measurements.


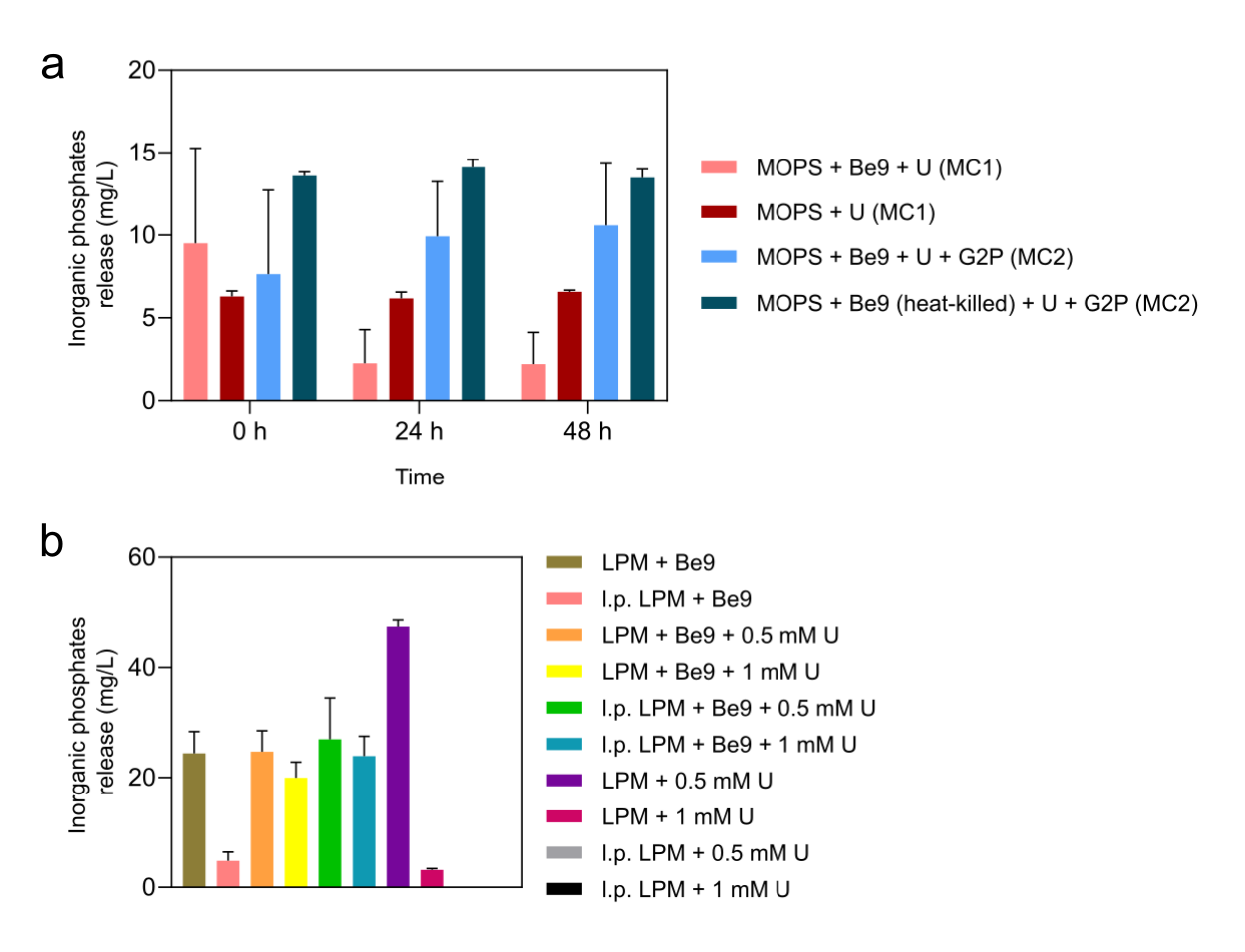


**Figure Supplementary 2.** Inorganic phosphates in solution (mg/L) detected during (A) MC1 and MC2 treatments incubation at different times (0, 24 and 48 h), and (B) MC3 treatment incubations at 48 h. Flasks without Be9 cells and heat-killed Be9 cells were used as control treatments. Data are showed as the mean ±SD of at least three independent measurements.


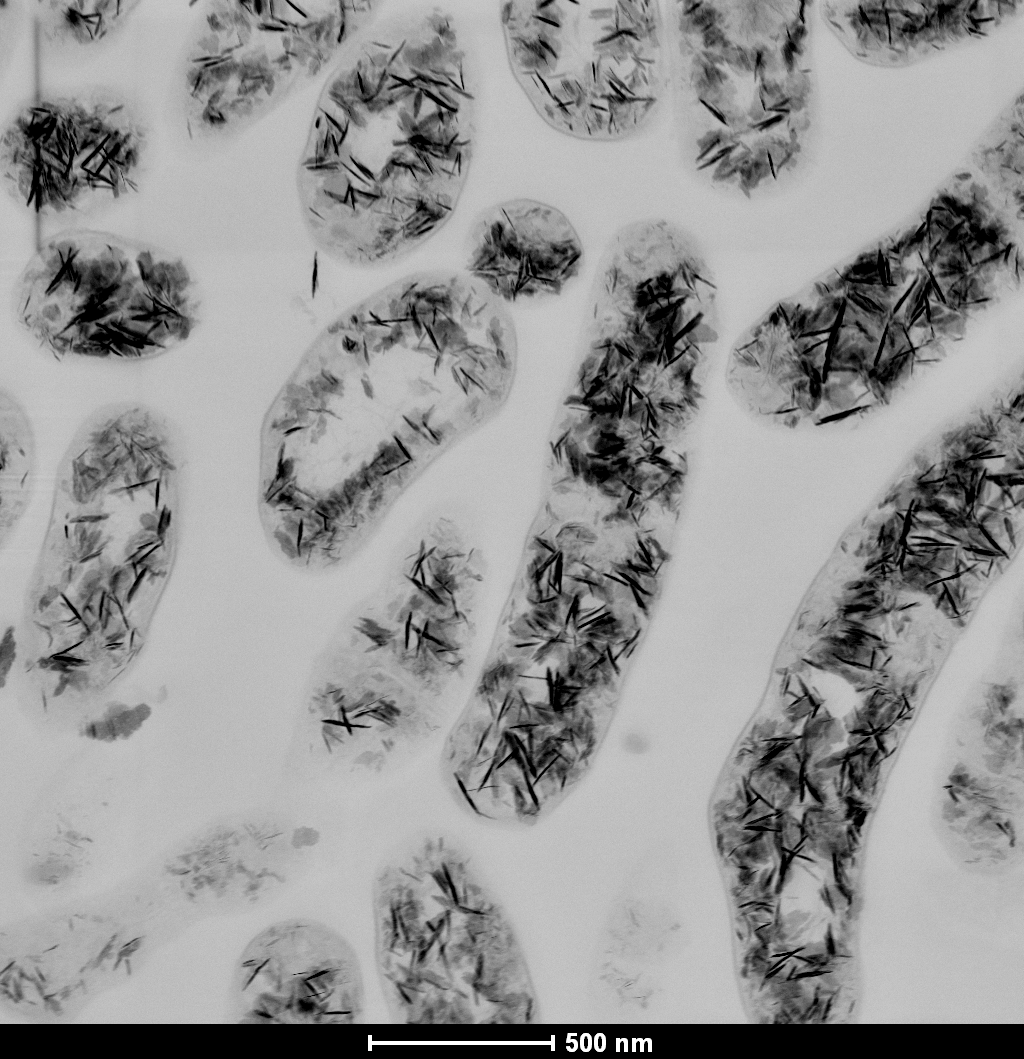


**Figure Supplementary 3.** HAADF-STEM image of a thin section of *Microbacterium* sp. Be9 cells recovered after their incubation in MOPS solution (5 mM) amended with U (0.1 mM). Intracellular U-precipitates are showed as condensed dark accumulations.

**Table Supplementary 4.** Cell viability and membrane potential of Be9 after 24 h and 48 h at different uranium concentrations. Data are showed as the mean and standard deviation is included as ± SD.

| **Conditions** | **Cell viability-24h** | | **Cell viability-48h** | | **Membrane potential-24h** | | **Membrane potential-48h** | |
| --- | --- | --- | --- | --- | --- | --- | --- | --- |
|  | **Alive (%)** | **Dead (%)** | **Alive (%)** | **Dead (%)** | **Active (%)** | **Non active (%)** | **Active (%)** | **Non active (%)** |
| **0 mM U** | 99.22 ± 0.22 | 0.78 ± 0.22 | 85.95 ± 0.26 | 14.05 **±** 0.26 | 99.18 **±** 0.07 | 0.82 **±** 0.07 | 99.00 **±** 0.00 | 1.00 **±** 0.00 |
| **0.1 mM U** | 100 ± 0.18 | 0.00 ± 0.18 | 18.42 ± 0.00 | 81.58 **±** 0.00 | 97.10 **±** 0.08 | 2.90 **±** 0.08 | 0.00 **±** 0.00 | 100 **±** 0.00 |


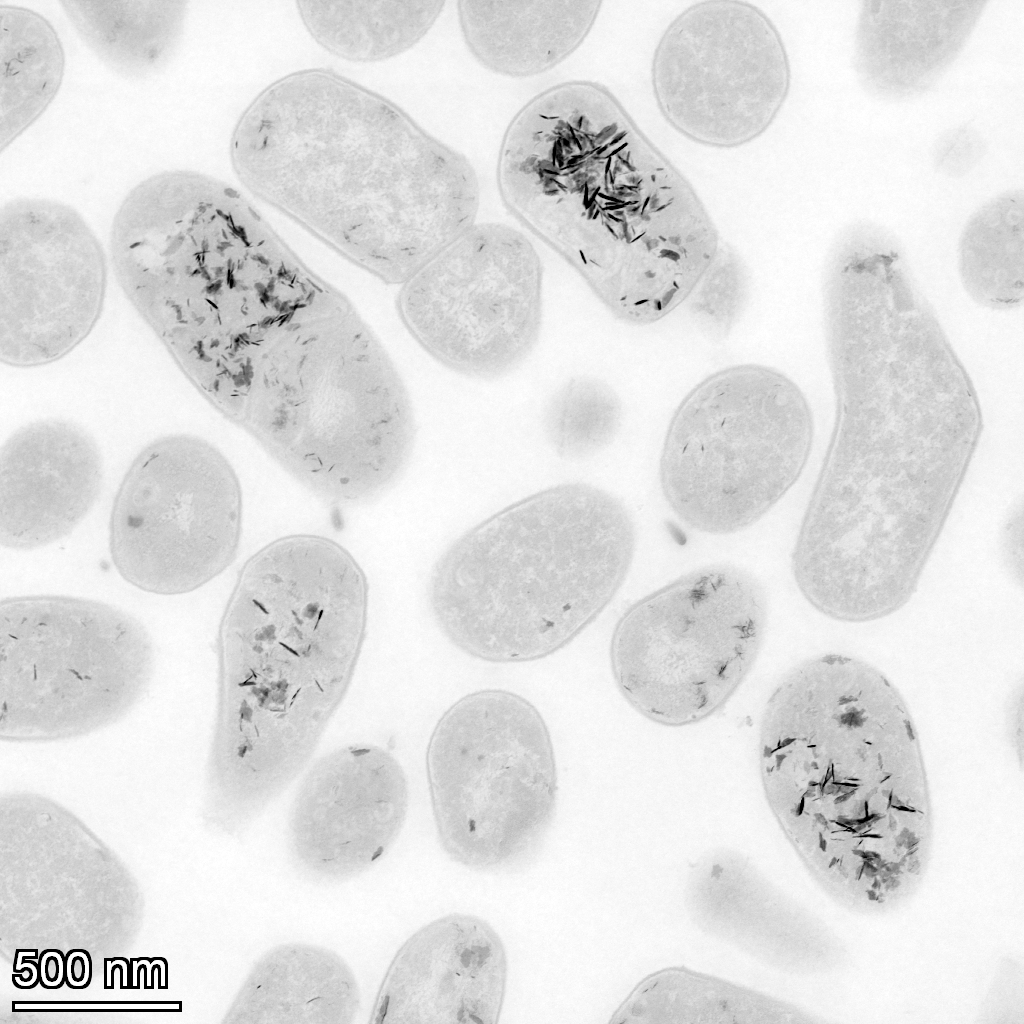


**Figure Supplementary 4.** HAADF-STEM image of a thin section of *Microbacterium* sp. Be9 cells recovered after their incubation in MOPS solution (5 mM) amended with U (0.1 mM) and G2P (5 mM). Intracellular U-precipitates are showed as condensed dark accumulations.





**Figure Supplementary 5**. HAADF-STEM image of U-precipitates formed abiotically after their incubation (48 h) in LPM amended with U (1 mM). U-precipitates are showed as condensed dark aggregates and needle-like fibrils.





**Figure Supplementary 6.** HAADF-STEM image of a thin section of *Microbacterium* sp. Be9 cells recovered after their incubation during 48 h in LPM amended with U (1 mM). Extracellular U-precipitates are showed as condensed dark accumulations.


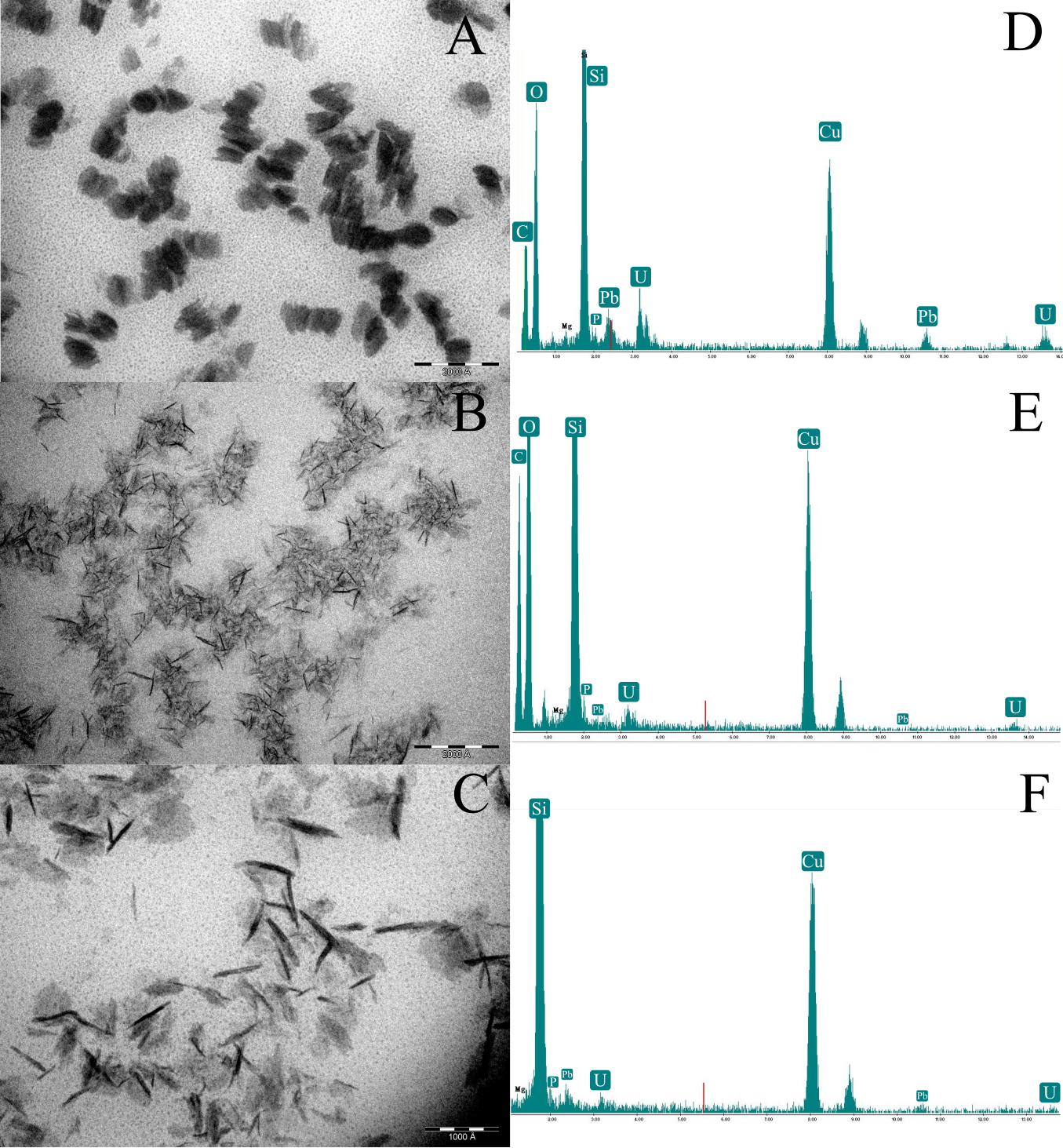


**Figure Supplementary 7.** HR-TEM images of (A) U (1 mM) amended abiotic low-peptone LPM sample, (B) U (0.1 mM) amended abiotic LPM sample, (C) U (1 Mm) amended abiotic LPM sample, and their respectively EDX analysis spectrum (D, E and F).
